# Intermittent spontaneous CSF rhinorrhea exacerbated by hormonal fluctuations associated with menstrual cycles

**DOI:** 10.1002/ccr3.9499

**Published:** 2024-10-21

**Authors:** Shriya P. Bhat, Allison Wyll, Amol Bhatki

**Affiliations:** ^1^ Molecular and Cellular Biology Harvard University Cambridge Massachusetts USA; ^2^ Department of Otolaryngology North Dallas ENT Dallas Texas USA; ^3^ Department of Otolaryngology, Head and Neck Surgery Baylor University Medical Center Dallas Texas USA

**Keywords:** CSF leak, female sex hormones, intermittent rhinorrhea, menstrual migraines

## Abstract

This case underscores the importance of recognizing the potential link between hormonal fluctuations during the menstrual cycle and idiopathic intracranial hypertension (IIH), which could lead to spontaneous cerebrospinal fluid (CSF) rhinorrhea. Eliciting a history of intermittent clear rhinorrhea with the onset of menstrual cycle if presenting with menstrual migraines could allow for a more prompt diagnosis and treatment of IIH and CSF leak. Early diagnosis and intervention are crucial to prevent serious complications.

## 1 INTRODUCTION

Cerebrospinal fluid (CSF) rhinorrhea, characterized by the leakage of CSF into the nose and sinuses, is a rare condition, with an average incidence of 0.001%.^1^ This usually occurs due to head trauma with an associated skull base fracture, leading to a communication between the subarachnoid space and the nasal cavity.^1^, ^2^ Symptomatology includes a persistent runny nose, headaches, nasal congestion, and complications such as meningitis and tension pneumocephalus.^2^ CSF fluid typically contains distinctive proteins, such as β‐2‐transferrin, that are not present in other bodily fluids like nasal fluid, and a positive β‐2‐transferrin test is considered the gold standard for CSF rhinorrhea diagnosis.^3^ While most CSF leaks are caused by closed‐head and penetrating trauma or previous surgical procedure, some cases (less than 5%) are spontaneous, with no known history of trauma or surgery. Of such cases, around 70% are associated with idiopathic intracranial hypertension (IIH).

Two known risk factors for IIH include obesity and female sex, with over 90% of patients affected by IIH being women.^4^ Female sex hormones have been implicated in intracranial pressure, with evidence suggesting intrinsic hormonal imbalance could be a risk factor and exogenous sex hormones affecting intracranial pressure.^5^, ^6^, ^7^, ^8^ Here, we report a rare case of intermittent spontaneous CSF rhinorrhea and menstrual migraines occurring at the onset of menstrual cycles in an obese female, which only became apparent after the CSF rhinorrhea became more persistent.

## 2 CASE HISTORY/EXAMINATION

A 44‐year‐old female with obesity, a history of chronic migraines, recurrent sinusitis, and allergic rhinitis presented to an outpatient clinic for a possible sinus infection. Her presenting symptoms included a 2‐month history of copious clear rhinorrhea, purulent postnasal drainage, and cough. The patient reported experiencing similar self‐limiting episodes of rhinorrhea during her menstrual cycle, preceded by episodes of severe menstrual migraines, which she thought were simply attributed to hormonal fluctuations of her menstrual cycle and therefore did not seek further medical attention. A 2‐week course of antibiotics, antihistamines, and steroid nasal spray for sinusitis and allergic rhinitis offered mild relief for the patient's cough—however, she continued to experience significant clear rhinorrhea, more copious on the left side. The patient responded poorly to a subsequent 4‐week trial of ipratropium nasal spray. She later reported developing a chronic headache several months prior to the onset of current symptoms, characterized by a severe, pounding sensation in the frontal and bi‐temporal areas, accompanied by tinnitus and visual disturbances. Notably, these headaches resolved with the onset of copious rhinorrhea.

**FIGURE 1:**
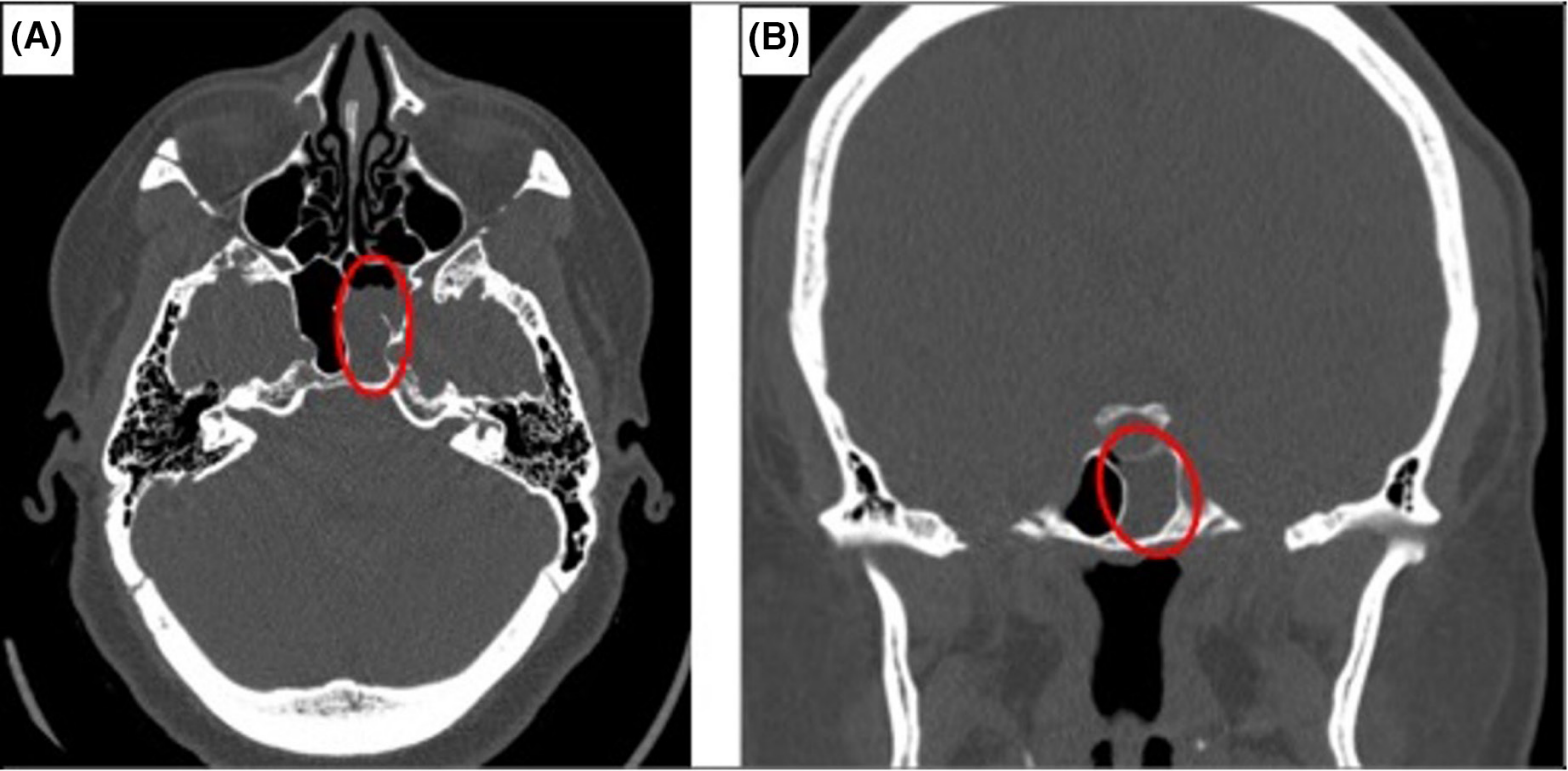
(A) Axial and (B) Coronal views of CT scan depict prominent air fluid level in the left sphenoid sinus extension (circled).

Otolaryngological examination was unremarkable upon anterior rhinoscopy, and the patient declined nasal endoscopy. Computed tomography (CT) scan revealed no notable masses, nasal obstructions, or obvious skull base defects, apart from an isolated sphenoid sinus disease (Figure 1). Upon further questioning, the patient reported that the nasal drainage was exacerbated by lifting, bending over, and waking up in the morning. This, coupled with prior history of intermittent self‐limiting rhinorrhea with the onset of menstrual period and history of headaches and pulsatile tinnitus which resolved upon the onset of copious unilateral clear rhinorrhea, prompted an assessment of the nasal drainage for cerebrospinal fluid (CSF).

## 3 METHODS (INVESTIGATION/DIFFERENTIAL DIAGNOSIS/TREATMENT)

**FIGURE 2:**
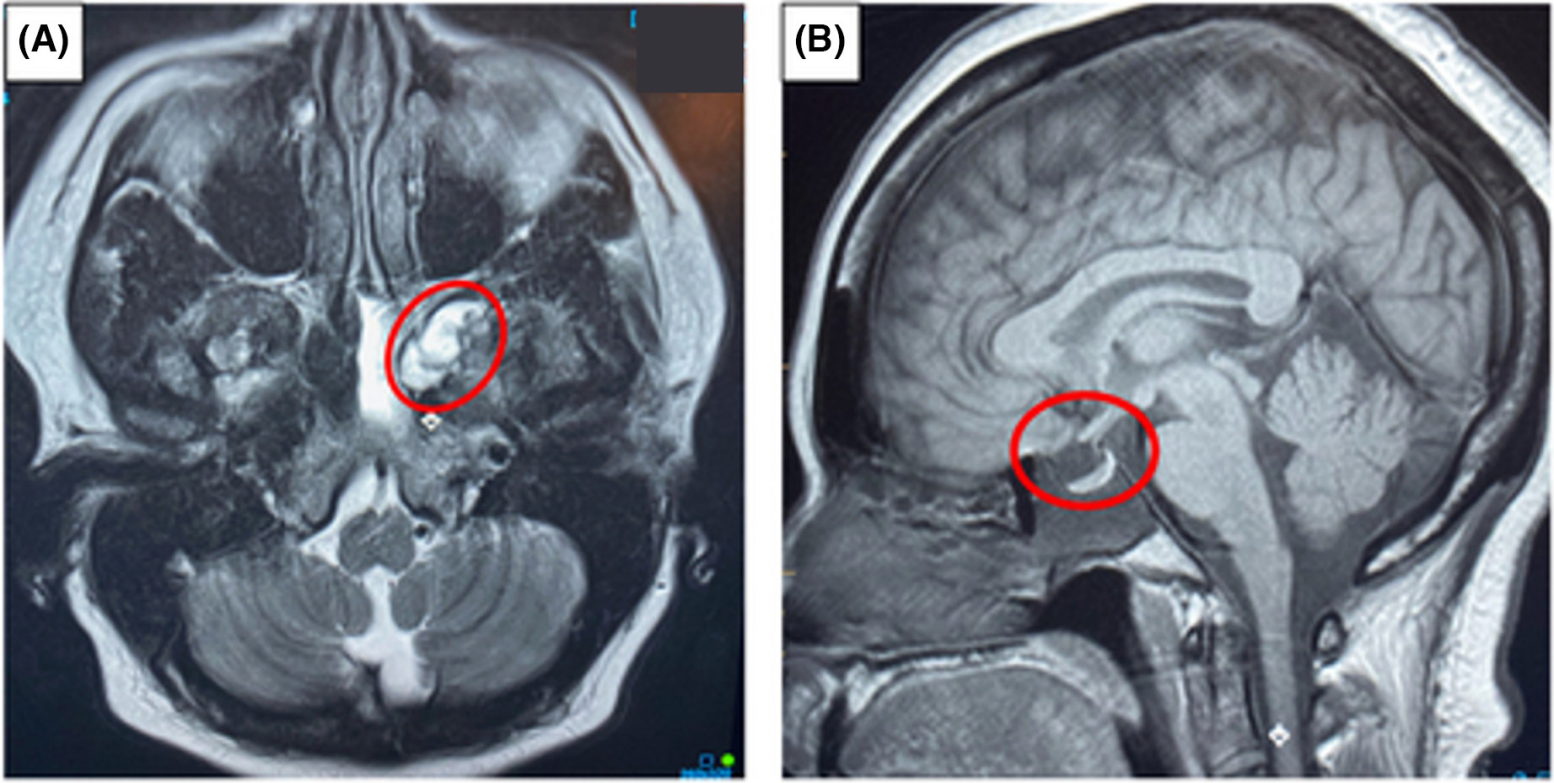
MRI of the skull base depicting (A) an encephalocele in the left sphenoid sinus (B) an empty sella (circled).

A positive β‐2 transferrin test of the nasal drainage confirmed the presence of CSF fluid, and therefore, in the absence of prior craniofacial trauma, sinus surgery, or neurosurgical procedure, we diagnosed spontaneous CSF rhinorrhea. Our patient was promptly referred to a skull base head and neck surgeon. The patient underwent magnetic resonance imaging (MRI) of the skull base which revealed an encephalocele in the left sphenoid sinus and a partial empty sella (Figure 2). Based on the clinical findings, IIH was thought to cause the CSF leak with the presumed defect in the lateral sphenoid recess on the left in the area of Sternberg's canal. Our patient then underwent an endoscopic endonasal resection of the temporal lobe encephalocele and repair of the skull base defect in the middle fossa. Repair was performed with a middle turbinate‐free mucosal graft.

## 4 CONCLUSION AND RESULTS (OUTCOME AND FOLLOW‐UP)

The patient experienced smooth postoperative recovery with no complications or residual nasal drainage. Since surgical repair of the CSF leak 8 weeks prior, the patient reports no copious rhinorrhea.

## 5 DISCUSSION

Spontaneous CSF rhinorrhea is rare, comprising about 5% of all CSF leak cases, of which IIH accounts for over 70% of cases.^3^ Our patient's history of rhinorrhea during her menstrual cycle was particularly noteworthy, as the symptoms were self‐limiting. The patient initially attributed the episodes of headaches preceding the rhinorrhea to menstrual migraines and therefore did not seek further medical attention. However, we hypothesize that these episodes of intermittent clear rhinorrhea were likely caused by a CSF leak exacerbated by increased intracranial pressure (ICP) associated with sharp decline in the levels of estrogen and progesterone that occurs before the onset of menses.^9^

Previous studies have found that transport capacity, and by extension CSF production, is reduced in rabbit choroid plexus pretreated with a combination of estrogen and progesterone, suggesting that sex hormones may influence ICP through direct action on the human choroid plexus.^9^, ^10^ The observation that combined estrogen and progesterone alter CSF production supports the hypothesis that hormonal fluctuations during the menstrual cycle could affect ICP and thus CSF leak as seen in our patient. During the luteal phase of the menstrual cycle, which occurs after ovulation, levels of both estradiol and progesterone are elevated. These levels drop sharply before menstruation and remain low throughout menses, with estradiol rising again leading up to ovulation.^11^ If estradiol and progesterone indeed affect CSF production in vivo, it is expected that CSF production would decrease during the luteal phase and increase just before and during menstruation. Thus, we hypothesize that the intermittent clear rhinorrhea preceded by severe headaches experienced by our patient at the onset of her menstrual cycles is associated with the transient increase in intracranial pressure due to the sudden decrease in estrogen and progesterone before her period. Additionally, estrogen has been shown to reduce brain edema and brain tissue water content, and pharmacological doses of both estrogen and progesterone improved ICP after brain injury, potentially suggesting the protective effect of estrogen and progesterone against IIH.^12^, ^13^

Spontaneous CSF rhinorrhea can be challenging to diagnose due to the lack of apparent precipitating cause: Common differential diagnoses are presented in Table 1.^14^ Obesity is also a major risk factor, likely contributing to the development of intracranial hypertension resulting in bone remodeling and thinning, and headaches presenting as menstrual migraines should heighten suspicion of IIH and possible risk of CSF rhinorrhea, especially in obese patients.^15^ Clinicians should remain vigilant for intermittent cases that can be challenging to diagnose as patients may not seek medical attention due to their self‐limiting nature and can be confounded by the presence of menstrual migraines.

**TABLE 1:** Differential diagnoses for patients presenting with clear rhinorrhea.

Differential diagnoses	Symptoms	Key distinguishing features
Spontaneous CSF rhinorrhea	Copious clear unilateral rhinorrhea	Reproducible by bending down, salty/sweet postnasal drainage, history of headaches and pulsatile tinnitus, history of OSA, poor response to antihistamines and ipratropium nasal spray; can be exacerbated by menstrual cycle
Traumatic CSF rhinorrhea	Copious clear unilateral or bilateral rhinorrhea	Reproducible by bending down, salty/sweet postnasal drainage, recent head trauma, history of sinus or neuro‐cranial surgery
Allergic rhinitis	Rhinorrhea, sneezing, nasal congestion, postnasal discharge, ocular and nasal itching	Bilateral symptoms, seasonal variation, good response to steroid nasal spray and antihistamines
Chronic rhinosinusitis with or without nasal polyps	Nasal congestion, sinus pressure, anterior or posterior nasal drainage, hyposmia	Bilateral symptoms, usually responds well to antibiotics and steroids
Primary nasal cavity tumor or nasopharyngeal carcinoma	Unilateral sinus pressure/congestion, headache, epistaxis, diplopia, facial numbness, neck mass due to cervical lymph node metastasis	Responds poorly to antibiotics, usually elderly, history of Epstein–Barr virus or HPV, smoking, excess alcohol consumption

This case highlights the potential link between menstrual cycle hormonal fluctuations and intermittent CSF leaks in patients with IIH, emphasizing the importance of considering this diagnosis in obese women presenting with cyclical headaches and rhinorrhea, even when symptoms are self‐limiting. Additionally, any cases of persistent unilateral rhinorrhea, exacerbated by bending over and with a salty or sweet postnasal drip, should alert a clinician about a possible CSF leak. Early detection and surgical correction of CSF rhinorrhea carry a significantly better prognosis, reducing potentially severe complications, including meningitis, meningoencephalitis, chronic postural headaches due to intracranial hypotension, hearing loss, or pulsatile tinnitus.^16^

## AUTHOR CONTRIBUTIONS

**Shriya P. Bhat:** Conceptualization; writing – original draft; writing – review and editing. **Allison Wyll:** Investigation; methodology; writing – review and editing. **Amol Bhatki:** Methodology; writing – review and editing.

## FUNDING INFORMATION

The authors report no funding for this case report.

## CONFLICT OF INTEREST STATEMENT

The authors declare that they have no conflict of interests.

## CONSENT

Written informed consent was obtained from the patient to publish this report in accordance with the journal's patient consent policy.

## Data Availability

Data sharing is not applicable to this article as no new data were created or analyzed in this study.

## References

[1] Graupman P , Nussbaum ES , Patel PD . Preventing cerebral spinal fluid leakage following endoscopy through a burr hole using a novel watertight closure: technical note. Br J Neurosurg. 2021;37(6):1915‐1917. doi:10.1080/02688697.2021.1903392 33779446

[2] Svider PF , Baredes S , Eloy JA . Pitfalls in sinus surgery: an overview of complications. Otolaryngol Clin N Am. 2015;48(5):725‐737. doi:10.1016/j.otc.2015.05.002 26117302

[3] Pérez MA , Bialer OY , Bruce BB , Newman NJ , Biousse V . Primary spontaneous cerebrospinal fluid leaks and idiopathic intracranial hypertension. J Neuroophthalmol. 2013;33(4):330‐337. doi:10.1097/WNO.0b013e318299c292 24042170 PMC4040082

[4] Chen J , Wall M . Epidemiology and risk factors for idiopathic intracranial hypertension. Int Ophthalmol Clin. 2014;54(1):1‐11. doi:10.1097/IIO.0b013e3182aabf11 PMC3864361 24296367

[5] Sheehan JP . Hormone replacement treatment and benign intracranial hypertension. Br Med J (Clin Res Ed). 1982;284(6330):1675‐1676. doi:10.1136/bmj.284.6330.1675 PMC1498610 6805652

[6] Abdelghaffar M , Hussein M , Abdelkareem SA , Elshebawy H . Sex hormones, CSF and serum leptin in patients with idiopathic intracranial hypertension. Egypt J Neurol Psychiatry Neurosurg. 2022;58:39. doi:10.1186/s41983-022-00473-x

[7] Markey KA , Uldall M , Botfield H , et al. Idiopathic intracranial hypertension, hormones, and 11β‐hydroxysteroid dehydrogenases. J Pain Res. 2016;9:223‐232. doi:10.2147/JPR.S80824 27186074 PMC4847593

[8] Hernández Castro I , López‐Rivera F . Medroxyprogesterone‐induced idiopathic intracranial hypertension in a non‐obese woman with a negative funduscopic examination. J Clin Images Med Case Rep. 2019;5:1. doi:10.5348/100049z09ic2019cr

[9] Lindvall‐Axelsson M , Owman C . Changes in transport functions of isolated rabbit choroid plexus under the influence of oestrogen and progesterone. Acta Physiol Scand. 1989;136(1):107‐111. doi:10.1111/j.1748-1716.1989.tb08635.x 2549764

[10] Lindvall‐Axelsson M , Owman C . Actions of sex steroids and corticosteroids on rabbit choroid plexus as shown by changes in transport capacity and rate of cerebrospinal fluid formation. Neurol Res. 1990;12(3):181‐186. doi:10.1080/01616412.1990.11739940 1979849

[11] Yen SSC , Vela P , Rankin J , Littell AS . Hormonal relationships during the menstrual cycle. JAMA. 1970;211(9):1513‐1517. doi:10.1001/jama.1970.03170090029006 5467048

[12] Naderi V , Khaksari M , Abbasi R , Maghool F . Estrogen provides neuroprotection against brain edema and blood brain barrier disruption through both estrogen receptors α and β following traumatic brain injury. Iran J Basic Med Sci. 2015;18(2):138‐144. 25810887 PMC4366724

[13] Shahrokhi N , Khaksari M , Soltani Z , Mahmoodi M , Nakhaee N . Effect of sex steroid hormones on brain edema, intracranial pressure, and neurologic outcomes after traumatic brain injury. Can J Physiol Pharmacol. 2010;88(4):414‐421. doi:10.1139/y09-126 20555409

[14] Bhat SP , Wyll A , Kwon H . Rare case of poorly differentiated squamous cell cancer of HPV etiology in a nasal polyp presenting as chronic sinusitis. Proc (Baylor Univ Med Cent). 2024;37(4):666‐669. doi:10.1080/08998280.2024.2346054 PMC11188785 38910823

[15] Hannerz J , Ericson K . The relationship between idiopathic intracranial hypertension and obesity. Headache. 2009;49(2):178‐184. doi:10.1111/j.1526-4610.2008.01240.x 19222591

[16] Zlab MK , Moore GF , Daly DT , Yonkers AJ . Cerebrospinal fluid rhinorrhea: a review of the literature. Ear Nose Throat J. 1992;71(7):314‐317. doi:10.1177/014556139207100707 1505380

